# [RETRACTED ARTICLE]: THE ROLE OF SERUM CK-18, MMP-9 AND TIPM-1 LEVELS IN PREDICTING R0 RESECTION IN PATIENTS WITH GASTRIC CANCER

**DOI:** 10.1590/0102-672020200002e1531

**Published:** 2020-11-20

ABCD staff team communicates formal Retraction about the article below:

Peduk S, Dincer M, Tatar C, Ozer B, Kocakusak A, Citlak G Akinci M. THE ROLE OF SERUM CK-18, MMP-9 AND TIPM-1 LEVELS IN PREDICTING R0 RESECTION IN PATIENTS WITH GASTRIC CANCER. ABCD, arq. bras. cir. dig. 2018; 31( 4 ): e1401. DOI: https://doi.org/10.1590/0102-672020180001e1401.

Since it was proved it's duplicate publication that was available in another journal.

Peduk S, Tatar C, Dincer M, Ozer B, Kocakusak A, Citlak G, Akinci M, Tuzun IS. The Role of Serum CK18, TIMP1, and MMP-9 Levels in Predicting R0 Resection in Patients with Gastric Cancer. Dis Markers. 2018 Feb 5;2018:5604702. doi: 10.1155/2018/5604702. PMID: 29651326; PMCID: PMC5832075.

Osvaldo Malafaia, MD, PhD, Full Professor of Surgery

Editor-in-Chief ABCD

